# A highly specific fluorescent probe with facile pretreatment for rapid and accurate detection of sulfur dioxide residues in wolfberry (*Lycium barbarum L*.)

**DOI:** 10.3389/fphar.2026.1759685

**Published:** 2026-02-20

**Authors:** Yuanyuan Ge, Wei Chen, Lingling Jiang, Kunhui Sun, Guojing Liu, Yanfeng Liu, Yibao Jin, Ping Wang, Liang Zhang, Mingtong Zhang, Lan Ma, Xie-An Yu, Bing Wang

**Affiliations:** 1 Shenzhen Institute for Drug Control, Shenzhen, China; 2 Shenyang Pharmaceutical University, Shenyang, China; 3 Chengdu Institute for Drug Control, Chengdu, China; 4 Gansu Institute for Drug Control, Lanzhou, China; 5 Tsinghua Shenzhen International Graduate School, Shenzhen, China

**Keywords:** facile pretreatment, fluorescent probe, rapid determination, sulfur dioxide residues, wolfberry

## Abstract

**Introduction:**

Sulfur fumigation is a traditional technique for drying, pest control and mildew prevention in traditional Chinese medicine (TCM). However, excessive sulfur fumigation significantly impairs drug safety and clinical efficacy, rendering sulfur dioxide (SO_2_) residues in sulfur-fumigated TCM a growing concern. Conventional detection methods exhibit drawbacks in practical operation, such as complex procedures, long time consumption, low sensitivity and expensive instruments.

**Methods:**

Herein, a fluorescent probe-based method was established for the rapid and accurate detection of SO_2_ residues in wolfberry, leveraging the nucleophilic addition-elimination reaction between the probe and sulfite. Considering the practical application requirements for the quality testing of TCM, the pretreatment parameters including sample forms, extraction modes, extraction times with extraction solvent were optimized. The detection protocol was finalized as follows: samples were soaked for 15 min in HEPES buffer (20 mM, pH 7.4), and the extract was reacted with the fluorescent probe at 35 °C for 15 min, followed by detection on a microplate reader.

**Results:**

The detection limit (LOD) was 1.5 μM, and the limit of quantification (LOQ) was 5 μM. The average recovery rates at low, medium, and high spiked concentrations ranged from 89.5% to 100.8%, the corresponding detection method was further applied to actual samples.

**Discussion:**

Accordingly, this method features facile pretreatment, rapid operation and detection, high accuracy and sensitivity, enabling rapid and reliable determination of SO_2_ residues in wolfberry. It provides a novel technical tool for TCM quality supervision and offers technical support for safeguarding public health.

## 1 Introduction

Wolfberry, derived from the dried ripe fruit of *Lycium barbarum L*., a plant belonging to the Solanaceae family (Pharmacopoeia Commission of the People’s Republic of China, 2025). It has multiple biological activities, including immunity enhancement, antioxidation, anti-aging, anti-tumor, blood lipid regulation, anti-inflammatory, antibacteria and eyesight-improving properties (Ilić et al., 2020; Wang et al., 2021; Xia et al., 2022; Yang et al., 2022; Lian et al., 2023; Li et al., 2023; Sanghavi et al., 2023; Han et al., 2025). As such, it is recognized as one of the classic traditional Chinese medicinal-edible dual-purpose herbs (Ma et al., 2023; Zhu et al., 2024). Given its significant medicinal and edible value, some manufacturers have resorted to excessive sulfur fumigation to artificially improve wolfberry’s appearance and extend its shelf life for economic benefits. Sulfur fumigation is a well-established traditional technique for the primary processing of medicinal materials (Deng et al., 2022). Although sulfur fumigation effectively eliminates insects, inhibits microbes, prevents mold growth, exerts a whitening effect and enhances product visual appearance (Zhan et al., 2018; Zhou et al., 2019; Guan et al., 2023; Jiang et al., 2023), it severely impairs the quality and safety of TCM by introducing sulfur dioxide residues (Xu et al., 2020; Liu et al., 2022). Excessive ingestion of these residues poses considerable health risks, including neurotoxicity, visceral damage and elevated carcinogenic risk (Sang et al., 2011; Jiang et al., 2020; Zhang et al., 2022; He et al., 2023). Thus, sulfur fumigation has aroused reflections and concerns regarding medication safety and public health.

To address this concern and ensure medication safety, the Chinese Pharmacopoeia has established specific limits for sulfur dioxide residues (Pharmacopoeia Commission of the People’s Republic of China, 2025). Current analytical techniques for detecting these residues include acid-base titration, ion chromatography, gas chromatography (Pharmacopoeia Commission of the People’s Republic of China, 2025), Raman spectroscopy (Kong et al., 2021) and electrochemical sensing (Manusha and Senthilkumar, 2020). However, these methods are often hampered by limitations such as operational complexity, reliance on costly instrumentation, prolonged analysis times for large sample batches and high operational skill requirements (Wang et al., 2025). Consequently, conventional detection techniques are inadequate to address the rapid response needs in modern pharmaceutical supply chain and regulatory practices (Zhang et al., 2025), there is a pressing need to develop detection methods that are faster, simpler to operate, and capable of high-throughput analysis (Zhao et al., 2024).

**SCHEME 1:**
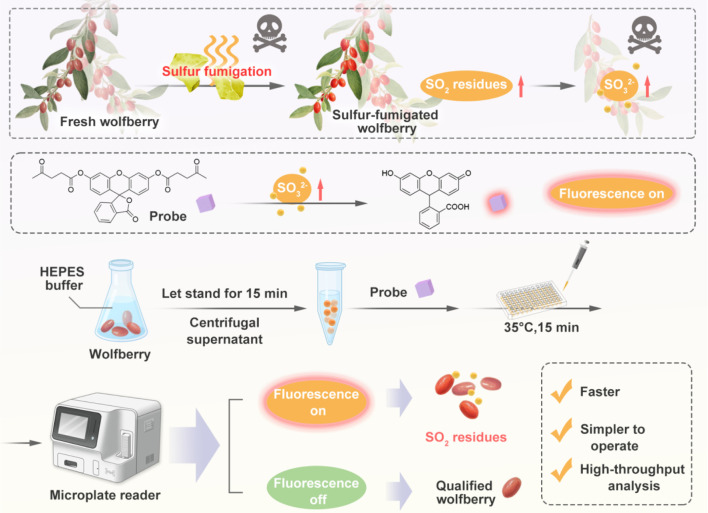
Schematic diagram of the fluorescent probe reacting with SO_3_^2-^ extracted from wolfberry to detect the sulfur dioxide residues.

In this study, a SO_2_-responsive fluorescent probe bearing a double cysteine residue was synthesized (Ma et al., 2013), which undergoes a nucleophilic addition-elimination reaction with the sulfite to yield a product with a luminescent moiety, enabling the quantitative detection of sulfite. Meanwhile, in practical applications, considering the complex matrix and numerous components of TCM, key pretreatment parameters including sample form, extraction mode, extraction solvent and extraction time were optimized. Eventually, a novel analytical method for determining SO_2_ residues was successfully developed and applied to wolfberry, exhibiting notable advantages in simplicity, rapidly and selectivity (Scheme 1). Overall, coupled with its advantages of simple sample pretreatment, short analysis time, and capability for rapid multi-batch detection, this method establishes itself as a highly valuable tool that merits comprehensive validation and broader implementation in TCM quality control.

## 2 Experimental section

### 2.1 Instruments and materials

The sample was weighed on electronic balance (XSR204, Mettler Toledo, Greifensee, Switzerland). FL spectra were acquired using a Varioskan Flash multimode microplate reader (3001, Thermo Fisher Scientific, Vantaa, Finland). The reaction system was maintained at in a biochemical incubator (KB53, BINDER GmbH, Tuttlingen, Germany). Supernatant was obtained in constant temperature large capacity centrifuge (DL-5-B, Shanghai anting scientific instrument factory, Shanghai, China). Ultrapure water (18.2 MΩ cm) was prepared using a Millipore Simplicity water purification system (Merck Millipore, MA, United States). Mass spectrometric analysis was conducted using an electrospray ionization (ESI) equipped X500R quadrupole time-of-flight (QTOF) mass spectrometer (AB Sciex, Framingham, MA, United States). The data were processed using Origin 2024 (OriginLab Corporation, Northampton, MA, United States).

The wolfberries were purchased from distinct manufacturers. SO_2_ fluorescent probe was obtained from Suzhou Zhuoxin Yayi Technology Co., Ltd. (Jiangsu, China). Sodium sulfite anhydrous (Na_2_SO_3_, ≥98.0% purity) was purchased from Shanghai Lingfeng Chemical Reagent Co., Ltd. (Shanghai, China). HPLC-grade acetonitrile (CH_3_CN, ≥99.9%) was acquired from Macklin Biochemical Co., Ltd. (Shanghai, China). HEPES buffer (20 mM, pH 7.4) was acquired from Shanghai Yuanye Bio-Technology Co., Ltd. (Shanghai, China). Anionic reagent was purchased from Shanghai Aladdin Biochemical Technology Co., Ltd. (Shanghai, China).

### 2.2 Solutions preparation and fluorescence detection system

The fluorescent probe was dissolved in acetonitrile as stock solution (2.0 mM). Stock solutions of common anions (234 mM) including sulfite and other typical anions (Cl^−^, HCO_3_^−^, H_2_PO_4_^−^, S_2_O_3_^2-^, F^−^, Br^−^, I^−^, SO_4_^2-^, PO_4_^3-^, NO_3_^−^, C_2_O_4_^2-^, CH_3_COO^−^, NO_2_^−^, HSO_3_^−^) were prepared by dissolving corresponding salts in HEPES buffer (20 mM, pH 7.4).

Varying concentrations of sulfite were mixed with 3 μL of the probe (60 μM) in 1.5 mL EP tubes. The volume was brought to 100 μL with HEPES buffer (20 mM, pH 7.4), and the solution was incubated at 35 °C for 15 min. Fluorescence intensity (λex/λem = 495/516 nm) was recorded on an enzyme label analyzer. A blank solution devoid of SO_3_^2-^ was prepared and measured concurrently. Each assay was replicated three times (n = 3).

### 2.3 Wolfberry extraction and detection

Specifically, 3 g of the sample was precisely weighed and mixed with 50 mL of HEPES buffer (20 mM, pH 7.4). The solution was allowed to stand for 15 min prior to centrifugation (5000 r/min, 5 min) to obtain the supernatant (test solution). For the detection assay, an aliquot of 5 μL of this test solution was combined with 3 μL of the probe (60 μM) in a 1.5 mL EP tube. The mixture was brought to a total volume of 100 μL with HEPES buffer, reacted at 35 °C for 15 min, and subsequently analyzed for fluorescence intensity (λex/λem = 495/516 nm) using an enzyme label analyzer.

## 3 Results and discussion

### 3.1 MS characterization of the fluorescent probe

**FIGURE 1:**
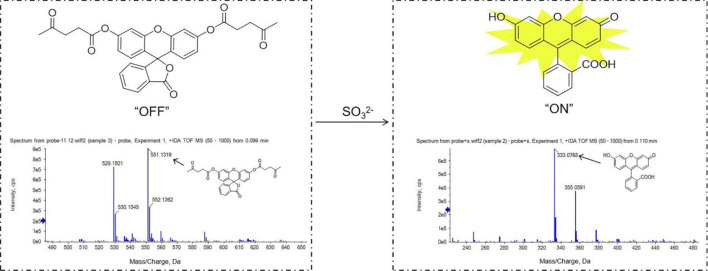
Mechanism of reaction between probe and SO_3_^2−^. IDA-TOF MS spectrum (positive ion mode) of fluorescent probe. [M + Na]^+^ calcd. for C_30_H_24_NaO_9_: 551.1313, found 551.1319. IDA-TOF MS spectrum (positive ion mode) of the raw solution (namely, probe + SO_3_^2-^) in a 15 min reaction time. [M + H]^+^ calcd. for C_20_H_13_O_5_: 333.0766, found 333.0768.

ESI-MS spectral analyses (positive ion mode) of the fluorescent probe solution (1 μg/mL) and the reaction solution of the probe with SO_3_^2-^ (100 mM) confirmed the proposed plausible mechanism. The plausible mechanism of fluorescent probe were shown in Figure 1. The peak of fluorescent probe at m/z 551.1319 (calcd m/z 551.1313 [M + Na]^+^), the peak of reaction product at m/z 333.0768 (calcd m/z 333.0763 [M + H]^+^).

The MS/MS spectra of the probe and reaction product were shown in Supplementary Figures S1, S2; Supplementary Table S1, the [M + Na]^+^ ion of probe at m/z 551.1305 was generated as the base peak, and ions at m/z 453.0937 [M-C_5_H_6_O_2_+Na]^+^, m/z 355.0569 [M-(C_5_H_6_O_2_)*2+Na]^+^ were observed. The [M + H]^+^ ion of reaction product at m/z 333.0766 served as the base peak, and ions at m/z 289.0860 [M-CHO_2_+H]^+^, m/z 271.0761 [M-CHO_2_-H_2_O + H]^+^ were observed.

### 3.2 Spectral properties and stability of the fluorescent probe

**FIGURE 2:**
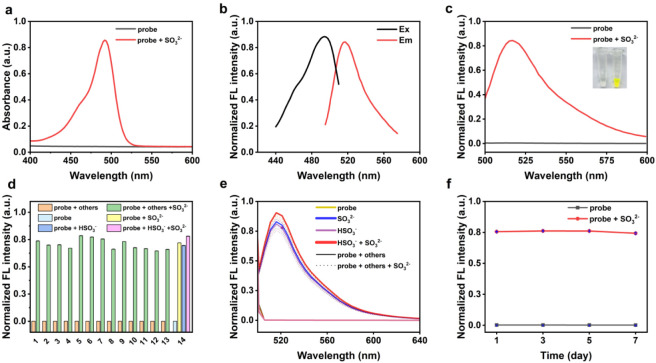
**(a)** The absorption spectra of probe (0.2 mM) in presence/absence of SO_3_^2−^ (2.34 mM). **(b)** Excitation and emission spectra of the probe. λex = 495 nm, λem = 516 nm. **(c)** Normalized fluorescence spectra of probe (0.2 mM) in presence/absence of SO_3_^2−^ (2.34 mM). Inset: photograph of probe before and after addition of SO_3_^2−^. Normalized fluorescence intensity **(d)** and spectra **(e)** of probe (0.2 mM) upon addition of various anions (2.34 mM), 1. Cl^−^. 2. HCO_3_^−^. 3. H_2_PO_4_^−^. 4. S_2_O_3_^2-^. 5. F^−^. 6. Br^−^. 7. I^−^. 8. SO_4_^2-^. 9.PO_4_^3-^. 10. NO_3_^−^. 11. C_2_O_4_^2-^. 12. CH_3_COO^−^. 13. NO_2_^−^. 14. HSO_3_^−^. **(f)** Storage stability of probe under −20 °C over 7 days.

The stable reaction system was established in the HEPES buffer (20 mM, pH 7.4). In the experiment, the probe (0.2 mM) was co-incubated with SO_3_^2-^ (2.34 mM) in HEPES buffer (20 mM, pH 7.4). First, one of the excitation or emission wavelengths was fixed, and the mixed solution was detected to obtain fluorescence intensity. The excitation and emission wavelengths were then screened based on the maximum fluorescence intensity. As shown in Figure 2b, the excitation and emission wavelengths were 495 nm and 516 nm, respectively. As shown in Figure 2c, the probe itself is non-fluorescent and colorless, upon addition of SO_3_^2-^ to the solution, it exhibits an enhanced fluorescence signal and a bright yellow color. Meanwhile, the results of ultraviolet absorption spectra (Figure 2a) and the visual color changes were mutually corroborative of each other.

Given the complexity of the TCM matrices, the anti-interference capability of the probe was evaluated by detecting various common anions, including Cl^−^, HCO_3_^−^, H_2_PO_4_^−^, S_2_O_3_^2-^, F^−^, Br^−^, I^−^, SO_4_^2-^, PO_4_^3-^, NO_3_^−^, C_2_O_4_^2-^, CH_3_COO^−^, NO_2_^−^ and HSO_3_^−^. The results indicated that the probe specifically recognizes SO_3_^2-^ with negligible response to other anions (Figures 2d,e). Overall, the probe showed high selectivity for SO_3_^2-^/HSO_3_^−^, supporting its applicability to sulfite detection in complex TCM matrices.

Next, the storage stability was evaluated by examining the probe stock solution after storage at −20 °C for 1, 3, 5 and 7 days. The relative standard deviation (RSD) of the fluorescence intensity was 2.95%, demonstrating that the probe exhibits reliable storage stability for up to 7 days (Figure 2f).

### 3.3 Optimization of probe detection conditions

**FIGURE 3:**
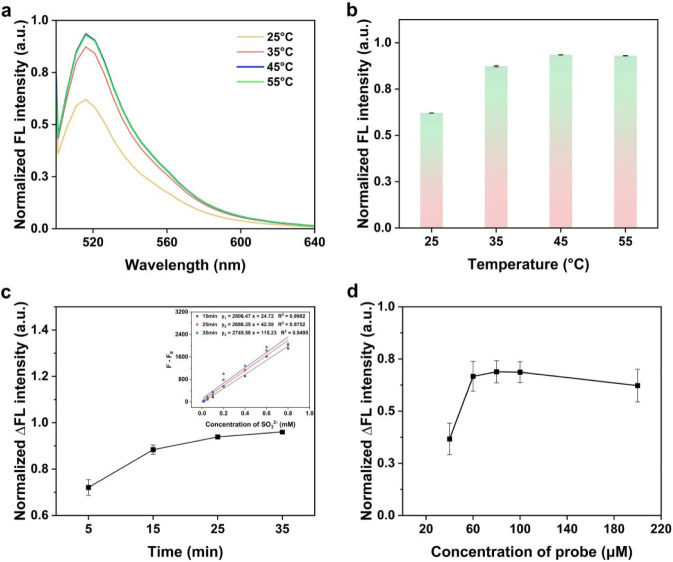
Normalized fluorescence spectra **(a)** and intensity **(b)** of probe (0.2 mM) with SO_3_^2-^ (2.34 mM) at different temperatures (25 °C–55 °C). **(c)** Normalized fluorescence intensity difference of probe (0.2 mM) with the SO_3_^2-^-containing (0.8 mM) system relative to the SO_3_^2-^-free blank system over time. Inset: Linear calibration relationship between SO_3_^2-^ concentration and normalized fluorescence intensity difference at 15, 25 and 35 min. **(d)** Normalized fluorescence intensity difference of the 7 μM SO_3_^2-^-containing system relative to the SO_3_^2-^-free blank system at various probe concentrations (40, 60, 80, 100, 200 μM).

Reaction temperature, incubation time and probe concentration were optimized to achieve faster, more complete and more sensitive detection of SO_3_^2-^. The study primarily verified the temperature effect. Data indicate that the reaction between the probe and SO_3_^2-^ tends toward completion at 35 °C (Figures 3a,b). Furthermore, the optimal reaction time was examined by assessing the reaction at time points of 5, 15, 25 and 35 min. Results showed that 15 min was sufficient for the detection system to react completely. The standard curve showed a better goodness of fit within 15 min (Figure 3c). Finally, the concentration of fluorescent probe was screened by fixing the amount of SO_3_^2-^. As indicated in Figure 3d, through the fluorescence intensity detection of the probe-SO_3_^2-^ reaction, 60 μM was determined to be the optimal probe concentration in the tested range (40, 60, 80, 100, 200 μM).

### 3.4 Optimization of wolfberry extraction conditions

To improve the accuracy and reliability of detection results, the key parameters of the sample extraction process were optimized with the recovery rate and RSD as the evaluation indices. The SO_3_^2-^ concentrations for the low, medium and high spiking levels were 7 μM, 25 μM and 50 μM. The 7 μM SO_3_^2-^ was derived by integrating the sample extraction process, with reference to the Chinese Pharmacopoeia, which stipulates that the sulfur dioxide residues should not exceed 150 mg/kg.

Moreover, matrix interference was observed in the sample extraction solution during fluorescence probe detection experiments, and the addition of 50 mL of this extraction solution mitigated such interference (Supplementary Figure S3). Meanwhile, there was no significant difference between the standard curve constructed in HEPES buffer (20 mM, pH 7.4) and that constructed in mixed blank wolfberry matrix (Supplementary Figure S4).

**FIGURE 4:**
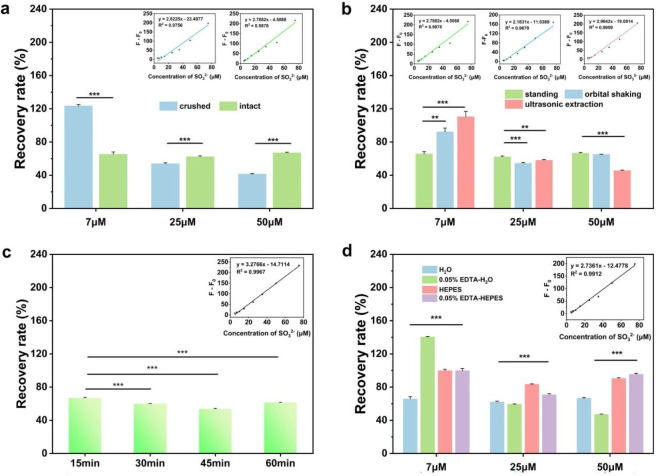
**(a)** Recovery of SO_3_^2-^ in different sample forms. Inset: Linear calibration relationship between SO_3_^2-^ concentration and fluorescence intensity difference for the crushed form (blue) and intact form (green). **(b)** Recovery of SO_3_^2-^ using different extraction methods. Inset: Linear calibration relationship between SO_3_^2-^ concentration and fluorescence intensity difference for the standing (green), orbital shaking (blue) and ultrasonic extraction (pink) methods. **(c)** Recovery of SO_3_^2-^ at different extraction times. Inset: Linear calibration relationship between SO_3_^2-^ concentration and fluorescence intensity difference at 15 min. **(d)** Recovery of SO_3_^2-^ using different extraction solvents. Significance analysis for the results of HEPES group compared to the other three groups respectively. Inset: Linear calibration between SO_3_^2-^ concentration and fluorescence intensity difference in HEPES buffer. **P < 0.01, ***P < 0.001.

The pretreatment parameters considered included sample forms (whole wolfberry, powder) and extraction conditions such as extraction modes (static, shaking, ultrasonic), extraction time, and extraction solvent. As shown in Figure 4a, the recovery rates of intact wolfberries spiked with low, medium, and high concentrations of SO_3_^2-^ were all above 60%. Additionally, Table 1 indicated that the RSD of the average recovery rates for the three concentrations in intact wolfberries was much lower than in powdered samples. Thus, subsequent experiments were conducted using intact wolfberries for further optimization.

**TABLE 1:** Optimization of pretreatment method for detection of SO_2_ residues in wolfberry.

Type		Recovery rate (%)			Average recovery rate (%)	RSD%
		Added (μM)				
		7	25	50		
Sample forms	Crushed	121.89	53.28	40.79	72.96	52.5
		125.02	55.18	42.01		
		123.58	53.30	41.57		
	Intact	68.35	63.43	67.81	64.80	3.9
		63.01	62.15	66.24		
		64.28	61.27	66.67		
Extraction modes	Orbital shaking	88.03	53.48	65.27	70.62	24.2
		92.18	54.53	64.96		
		96.82	55.12	65.23		
	Ultrasonic	117.51	59.07	46.26	71.36	42.1
		108.96	57.27	45.27		
		105.05	57.57	45.29		
	Standing	68.84	63.22	67.42	64.78	4.0
		63.54	61.96	65.87		
		64.80	61.08	66.30		
Extraction solvent	H_2_0	68.35	63.43	67.81	64.80	3.9
		63.01	62.15	66.24		
		64.28	61.27	66.67		
	0.05% EDTA-H_2_O	139.93	59.76	47.27	82.31	53.4
		140.83	59.33	47.31		
		140.78	58.97	46.59		
	HEPES	101.44	82.91	89.41	91.21	7.9
		99.92	83.96	91.40		
		98.12	83.33	90.44		
	0.05% EDTA-HEPES	97.74	69.29	96.25	88.65	15.4
		102.74	71.01	96.16		
		98.88	71.97	93.84		

Figure 4b showed that the recovery rates of low, medium and high concentrations of SO_3_^2-^ stabilized at around 65%. Table 1 showed that compared with the shaking and ultrasonic methods, the RSD of the average recovery rates for the three concentrations the static soaking method is lower, indicating that the static method is relatively stable across low, medium and high concentrations.

Figure 4c showed that 15 min was the optimal extraction time, as determined by comparing the recovery rates of 15, 30, 45 and 60 min. As shown in Figure 4d; Table 1, the HEPES buffer (20 mM, pH 7.4) was selected as the optimal extraction solvent because it yielded higher recovery rates and a relatively lower RSD compared with other solvents.

In addition, the fluorescence intensity exhibited a linear correlation with SO_3_^2-^ concentration (*R* ^2^ = 0.9912, y = 2.7361x-12.4778, 5–75 μM). The limit of detection (LOD = 3 σ/k, σ = 1.3532, k = 2.7361) was calculated to be 1.5 μM.

All the results confirmed that the optimal conditions were as follows: accurately weigh 3 g of sample, precisely add 50 mL of HEPES buffer (20 mM, pH 7.4), let stand for 15 min, centrifuged, and use the supernatant as the test solution. At the end of the experiment, the low, middle, high recovery rates of 10 batches of samples were detected using this method. As shown in Table 2, the average recovery rate ranged between 89.5% and 100.8%.

**TABLE 2:** Spiked recovery rates of 10 batches of wolfberries.

Added amount (μM)	Sample 1	Sample 2	Sample 3	Sample 4	Sample 5	Sample 6	Sample 7	Sample 8	Sample 9	Sample 10
Recovery rate (%)										
7.00	83.55	115.88	111.50	73.99	92.14	75.10	79.72	102.35	76.68	67.77
	84.20	116.45	123.97	70.63	95.85	71.10	82.95	97.45	84.77	71.54
	85.30	124.98	120.10	75.01	94.27	80.64	76.81	98.83	82.06	70.75
	Average recovery rate (%)									
	84.35	119.10	118.52	73.21	94.09	75.61	79.83	99.54	81.17	70.02
	RSD (%)									
	1.05	4.28	5.38	3.13	1.97	6.34	3.85	2.54	5.08	2.84
Recovery rate (%)										
24.99	106.48	119.74	99.98	94.70	79.72	108.63	82.00	98.20	89.02	94.38
	104.92	114.03	99.52	93.40	83.07	106.74	83.59	96.26	90.49	93.43
	99.86	115.44	98.95	95.06	83.27	108.90	82.93	97.05	89.97	96.62
	Average recovery rate (%)									
	103.75	116.40	99.48	94.38	82.02	108.09	82.84	97.17	89.83	94.81
	RSD (%)									
	3.33	2.56	0.52	0.93	2.43	1.09	0.96	1.00	0.83	1.73
Recovery rate (%)										
49.98	105.41	131.04	122.64	98.69	81.75	106.07	86.31	101.75	85.00	87.98
	105.07	130.20	123.20	100.08	81.96	107.66	86.58	101.32	84.72	87.91
	106.11	129.12	124.58	99.87	81.88	105.25	87.31	102.67	86.03	88.46
	Average recovery rate (%)									
	105.53	130.12	123.48	99.55	81.86	106.33	86.73	101.91	85.25	88.12
	RSD (%)									
	0.50	0.74	0.81	0.76	0.13	1.15	0.59	0.67	0.81	0.34

Finally, the practicality of the new method was verified by the synchronous detection of wolfberry using both the fluorescent probe method and the titration method, and the results showed that According to the limit value of sulfur dioxide residues in wolfberry specified in *the Pharmacopoeia of the People’s Republic of China*, the fluorescent probe method can be used to obtain the test results, on the basis of which the qualification of samples is determined. The detailed results are presented in Table 3.

**TABLE 3:** The detection results of wolfberry were synchronously validated by the fluorescent probe method and the acid-base titration method (*the Pharmacopoeia of the People’s Republic of China*).

Sample	Sulfur dioxide residues (mg/kg)	
	Fluorescent probe method	Titration method
1	114.7	114.4
2	-	17.5
3	-	<10
4	167.9	160.3
5	208.1	192.5

LOQ (5 μM) corresponding to 106.7 mg/kg of SO_2_.

-:The concentration is less than 106.7 mg/kg. Indicating that the content does not exceed the limit of 150 mg/kg specified in the *Pharmacopoeia of the People’s Republic of China*.

## 4 Conclusion

Herein, a fluorescent probe-based method was established and validated for the rapid, accurate detection of SO_2_ residues in wolfberry. Collectively, endowed with the advantages of rapidity, high selectivity and colorimetric visualization, the developed fluorescent probe method is expected to cater to the needs of rapid screening at production sites and on-site regulatory inspections for SO_2_ residues in TCM. From a longer-term perspective, fluorescent probe technology will advance the modernization of exogenous toxicant detection in TCM by virtue of its merits: high specificity, high sensitivity, elimination of complex pretreatment, rapid response and non-destructive sample analysis. This research is anticipated to offer novel methodological and technical support for the quality assurance of TCM.

## Data Availability

The original contributions presented in the study are included in the article/Supplementary Material, further inquiries can be directed to the corresponding authors.
