# Supplementary material for: A highly specific fluorescent probe with facile pretreatment for rapid and accurate detection of sulfur dioxide residues in wolfberry (Lycium barbarum L.)
Source: Front Pharmacol. 2026 Feb 20;17:1759685. doi: 10.3389/fphar.2026.1759685 (PMC12963281; doi:10.3389/fphar.2026.1759685)
Supplement: Supplementary file 1 [file Supplementaryfile1.docx]

Supplementary Material

**A highly specific fluorescent probe with facile pretreatment for rapid and accurate detection of sulfur dioxide residue in Wolfberry ( *Lycium barbarum L*.)**

**Yuanyuan Ge^1,†^, Wei Chen^1,2†^, Lingling Jiang^1^, Kunhui Sun^1^, Guojing Liu^1^, Yanfeng Liu^2^, Yibao Jin^1^, Ping Wang^1^, Liang Zhang^4^, Mingtong Zhang^5^, Lan Ma^3,*^, Xie-an Yu^1,*^, Bing Wang^1,*^**

^1^Shenzhen Institute for Drug Control, Shenzhen, China

^2^Shenyang Pharmaceutical University, Shenyang, China

^3^Tsinghua Shenzhen International Graduate School, Shenzhen, China

^4^Chengdu Institute for Drug Control, Chengdu, China

^5^Gansu Institute for Drug Control, Gansu, China

^†^ These authors have contributed equally to this work

**^*^ Corresponding authors**: Lan Ma, Email: [malan@sz.tsinghua.edu.cn](mailto:malan@sz.tsinghua.edu.cn); Xie-an Yu, Email: yuxieanalj@126.com; Bing Wang, Email: wangbingszyj@163.com;

# Supplementary Data

**Supplementary Figures**

**
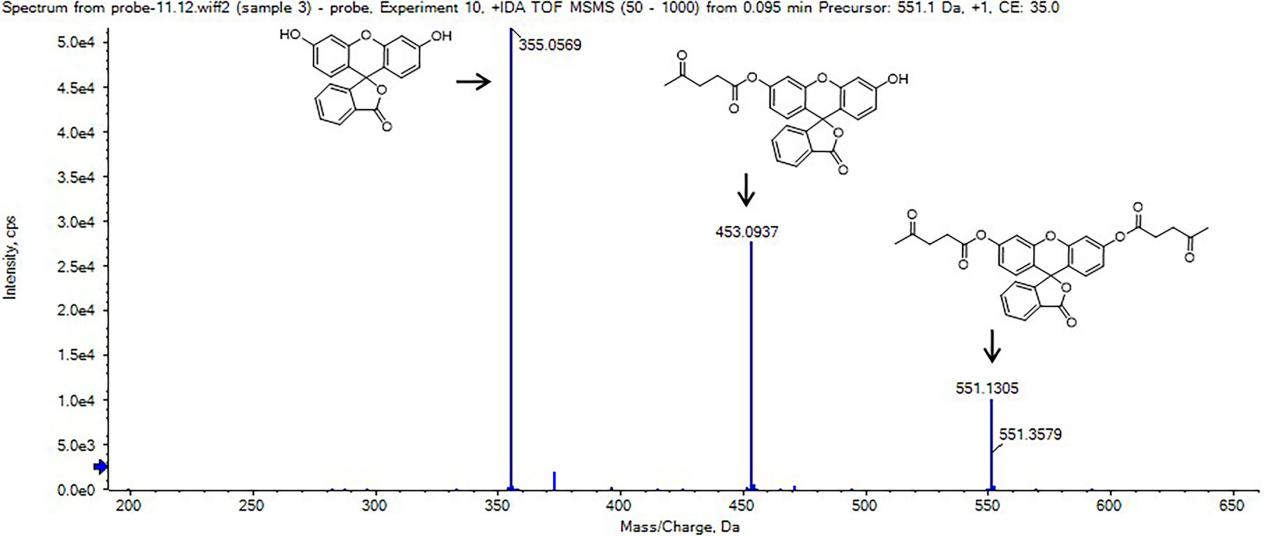
**

Fig. S1. IDA-TOF-MSMS spectrum (positive ion mode) of the fluorescent probe.


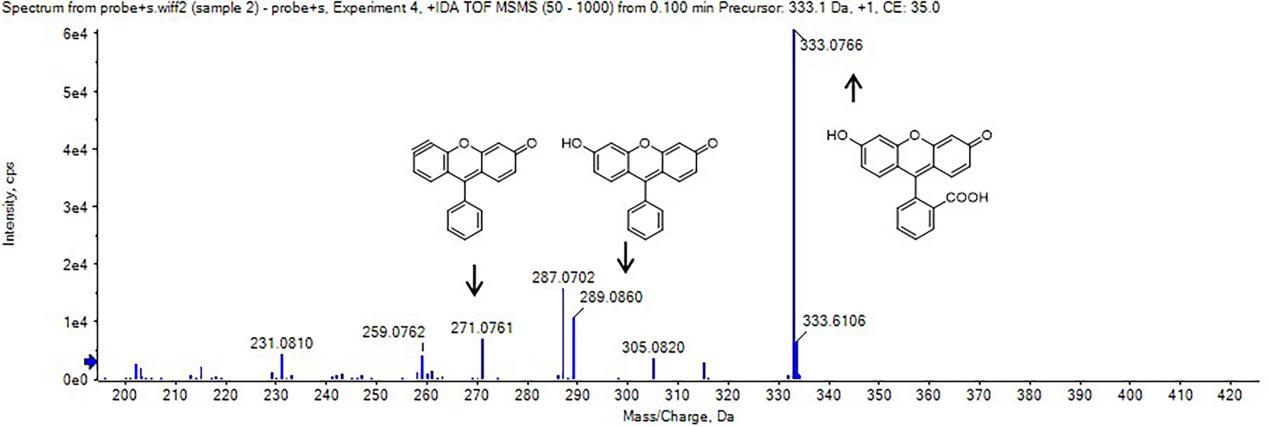


Fig. S2. IDA-TOF-MSMS spectrum (positive ion mode) of the raw solution (namely, probe + SO_3_^2−^) in a 15 min reaction time.


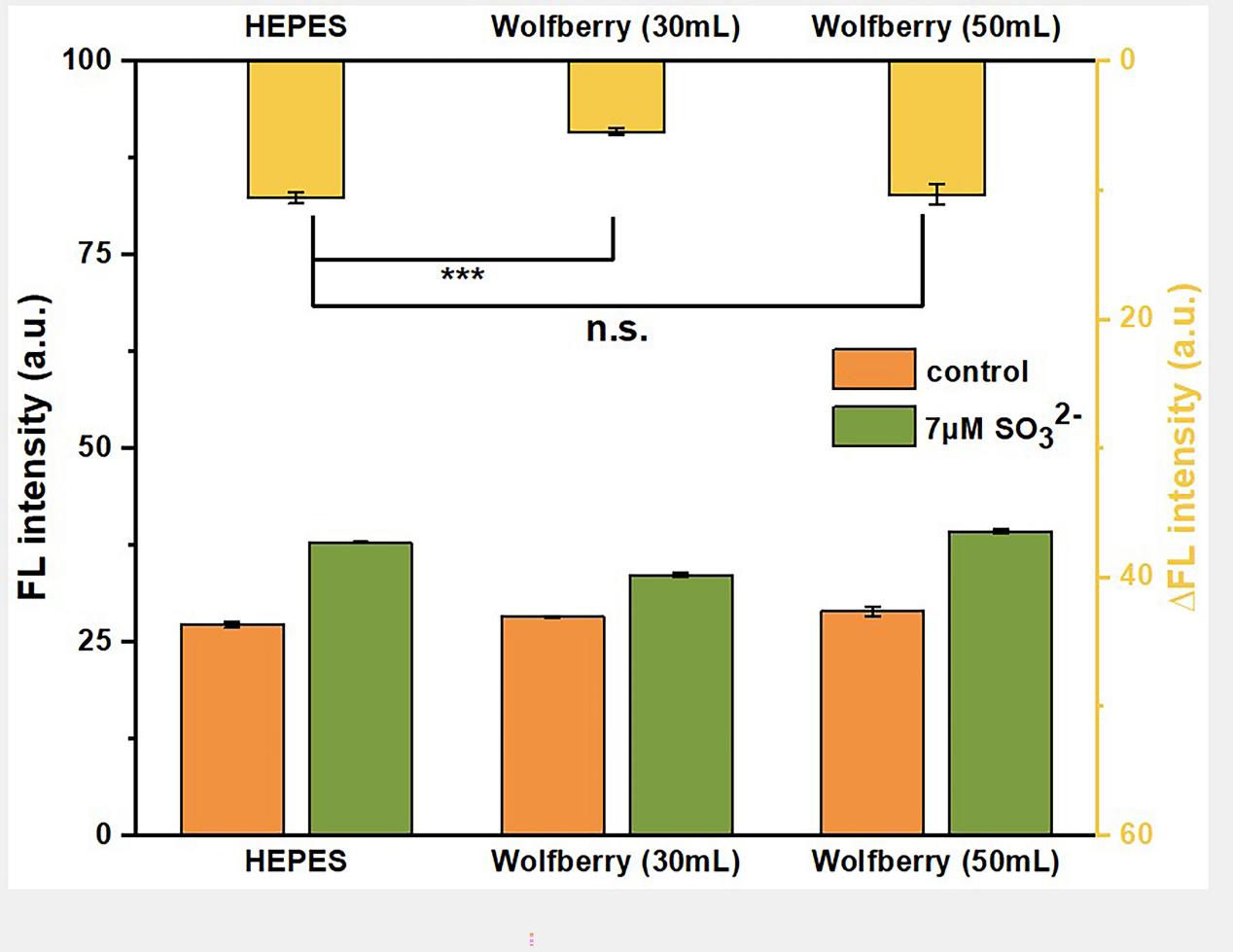


Fig. S3 Fluorescence intensities of SO_3_^2-^-free and 7 μM SO_3_^2-^ measured separately in HEPES buffer, 30 mL and 50 mL of wolfberry blank matrix extraction solution. n.s.: no significant, ***P< 0.001.


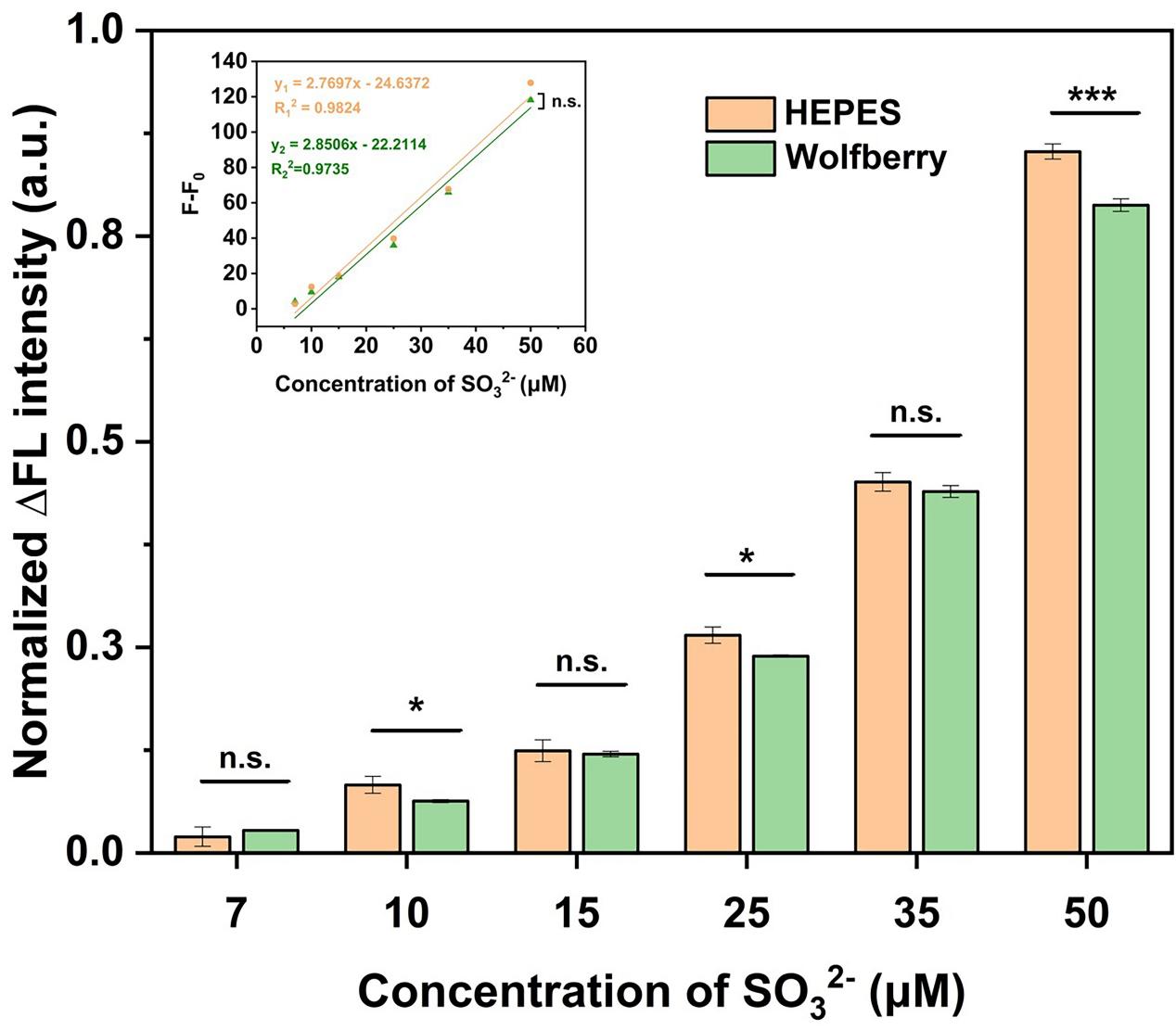


Fig. S4 Fluorescence intensity induced by different SO_3_^2-^ under two conditions: with (orange) and without (green) wolfberry matrix. The inset shows the linear relationship between the fluorescence signal difference under both conditions and sulfite concentration. n.s.: no significant, *P < 0.05, ***P< 0.001.


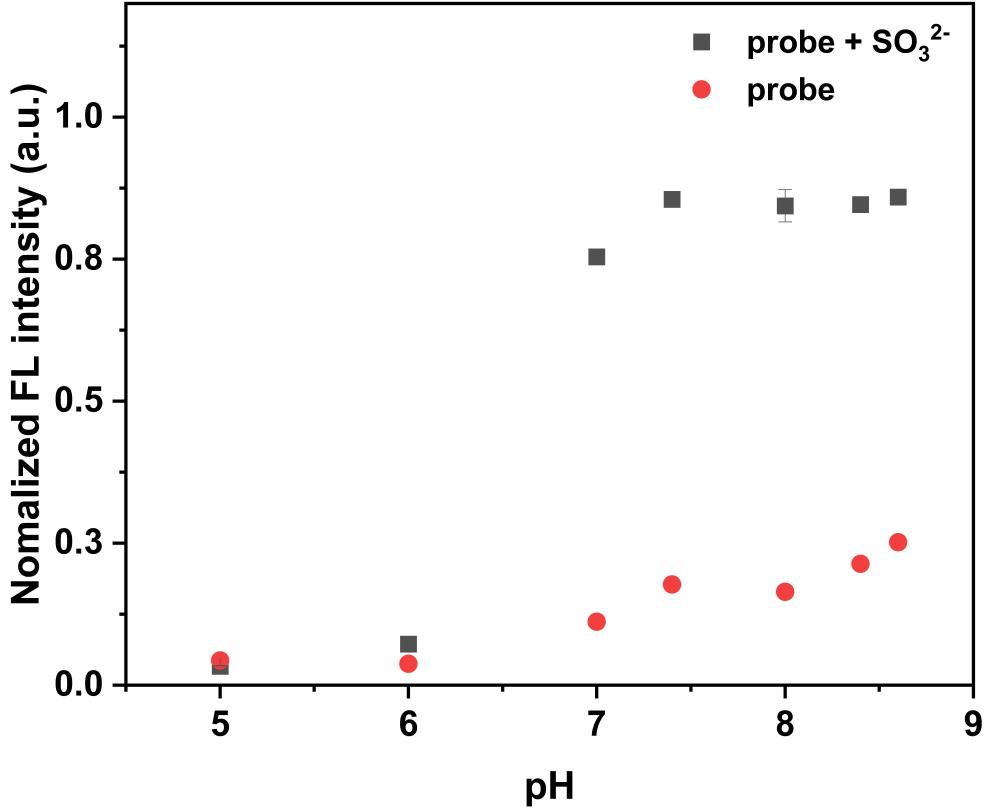


Fig. S5 The pH-dependent fluorescence intensity (**λex/λem = 495/516 nm**) for probe

(60 μM) and mixture of probe 1 (60 μM) and SO_3_^2−^ (50 μM) measured in various pH values at 35℃.

# Supplementary Tables

Table S1 Identification of the fluorescent probe and the reaction product of the probe with SO_3_^2-^

| Molecular formula | Ion type | Found at Mass | Error (ppm) | MS/MS fragments |
| --- | --- | --- | --- | --- |
| C_30_H_24_O_9_ | [M+Na]^+^ | 551.1305 | -0.3 | 453.0937 |
|  |  |  |  | 355.0569 |
| C_20_H_12_O_5_ | [M+H]^+^ | 333.0766 | 2.0 | 287.0702 |
|  |  |  |  | 271.0761 |
